# Relationship between periodontal disease and cardiovascular risk factors among young and middle-aged Brazilians. Cross-sectional study: Relação entre doença periodontal e fatores de risco cardiovascular em brasileiros jovens e de meia-idade. Estudo transversal

**DOI:** 10.1590/1516-3180.2016.0357300117

**Published:** 2017-04-03

**Authors:** Alessandra Carvalho Goulart, Favius Armani, Astrid Marie Arap, Thais Nejm, Juliana Barros Andrade, Henry Bittar Bufarah, Danielli Haddad Syllos Dezen

**Affiliations:** I MD, PhD. Clinical Epidemiologist, Health Promotion and Check-up Center, Hospital Sírio Libanês, São Paulo (SP); and Clinical Epidemiologist, Center for Clinical and Epidemiological Research, Hospital Universitário, Universidade de São Paulo (USP), São Paulo (SP), Brazil.; II DDS. Dentist, Health Promotion and Check-up Center, Hospital Sírio Libanês, São Paulo (SP), Brazil.; III MSc. Dentist, Health Promotion and Check-up Center, Hospital Sírio Libanês, São Paulo (SP), Brazil.; IV MD. Dentist, Health Promotion and Check-up Center, Hospital Sírio Libanês, São Paulo (SP), Brazil.

**Keywords:** Obesity, Periodontitis, Periodontal diseases, Cross-sectional studies, Risk factors

## Abstract

**CONTEXT AND OBJECTIVE:**

It has been suggested in the literature that periodontal disease (PD) is associated with cardiovascular risk. The objective of this study was to appraise the relationship between periodontal disease (gingivitis and periodontitis) and traditional cardiovascular risk factors (obesity, hypertension, dyslipidemia, diabetes and metabolic syndrome) among young and middle-aged adults attended at a health promotion and check-up center in the city of São Paulo, Brazil.

**DESIGN AND SETTING:**

Cross-sectional study at the Health Promotion and Check-up Center of Hospital Sírio-Libanês, São Paulo, Brazil.

**METHODS:**

We consecutively evaluated 539 subjects without prior cardiovascular disease who were seen within a health promotion program that included cardiovascular and dental evaluation between February and November 2012. Odds ratios (OR) with respective 95% confidence intervals (95% CI) for the association between PD and cardiovascular risk factors were ascertained through multinomial logistic regression.

**RESULTS:**

In this sample of mean age 45 years (standard deviation, SD ± 8.8), which was 82% male, we found PD in 63.2% (gingivitis 50.6% and periodontitis 12.6%). Individuals with PD were older, more obese (without PD 15.2%; versus gingivitis 22.1% and periodontitis 32.4%) and more diabetic (without PD 5.1%; versus gingivitis 4.8% and periodontitis 13.2%), compared with those without PD. Among all cardiovascular risk factors evaluated, obesity was associated with periodontitis (multivariate OR, 2.36; 95% CI, 1.23-4.52). However, after additional adjustment for oral hygiene, this finding was no longer significant (multivariate OR, 1.63; 95% CI, 0.79-3.37).

**CONCLUSIONS:**

We did not find any significant associations between cardiovascular risk factors and periodontal disease in this sample.

## RESUMO

**CONTEXTO E OBJETIVO:**

A literatura sugere que doença periodontal (DP) está associada ao risco cardiovascular. O objetivo deste estudo foi avaliar a relação entre doença periodontal (gengivite e periodontite) e fatores de risco cardiovasculares tradicionais (obesidade, hipertensão, dislipidemia, diabetes e síndrome metabólica) em adultos jovens e de meia-idade atendidos em um centro de promoção da saúde e *check-up* na cidade de São Paulo, Brasil.

**TIPO DE ESTUDO E LOCAL:**

Estudo transversal no Centro de Promoção de Saúde e *check-up* do Hospital Sírio-Libanês, São Paulo, Brasil.

**MÉTODOS:**

Avaliamos consecutivamente 539 indivíduos sem doença cardiovascular diagnosticada, observados em um programa de promoção da saúde que incluiu avaliação cardiovascular e odontológica de fevereiro a novembro de 2012. Razões de chances (RC) com respectivos intervalos de confiança de 95% (IC 95%) para a associação entre DP e fatores de risco cardiovascular foram calculadas por regressão logística multinomial.

**RESULTADOS:**

Nesta amostra de pessoas com idade média de 45 anos (desvio padrão, DP ± 8,8), 82% de homens, encontramos 63,2% de casos de DP (50,6% de gengivite e periodontite 12,6%). Indivíduos com DP eram mais velhos, mais obesos (sem DP 15,2%; *versus* gengivite 22,1% e periodontite 32,4%) e mais diabéticos (sem DP 5,1%; *versus* gengivite 4,8% e periodontite 13,2%) comparados com aqueles sem DP. De todos os fatores de risco cardiovascular avaliados, a obesidade foi associada à periodontite (RC multivariada, 2,36; IC 95%, 1,23-4,52). No entanto, após ajuste adicional para higiene bucal, esse achado não foi mais significativo (RC multivariada, 1,63; IC 95%, 0,79-3,37).

**CONCLUSÕES:**

Não encontramos associações significativas entre fatores de risco cardiovascular e doença periodontal nesta amostra.

## INTRODUCTION

Recent evidence has suggested that periodontal disease (PD) is an emerging risk factor for fatal and non-fatal cardiovascular outcomes.^1^^,^^2^^,^^3^^,^^4^^,^^5^Pooled data from a systematic review found that PD was independently associated with increased risk of coronary heart disease (CHD), with risk estimates ranging from 1.24 (95% confidence interval, CI, 1.01-1.51) to 1.34 (95% CI, 1.10-1.63).^1^

In the Normative Aging and Dental Longitudinal Study, which was conducted on 1,203 men who were followed up for 35 years, chronic periodontitis was also associated with increased incidence of CHD, particularly among individuals younger than 60 years of age, independent of established cardiovascular risk factors (CVRFs) such as dyslipidemia, diabetes and hypertension. In that study, a hazard ratio (HR) of 2.12 (95% CI, 1.26-3.60) from comparing the highest versus the lowest category of radiographic bone loss (which is a measurement of advanced PD) was reported for individuals younger than 60 years, but not for those older than 60 years.^2^ Moreover, PD is also associated with all-cause and cardiovascular disease (CVD) mortality.^3^^,^^4^ In the VA Normative Aging and Dental Longitudinal Study, for each 20% increase in mean whole-mouth radiographic alveolar bone loss (ABL), the risk of death increased by 51%.^3^

In the National Health and Nutrition Examination Survey III (NHANES III), prospective evaluations were performed on 10,849 participants, and 3,105 and 561 individuals were identified as having moderate and severe PD, respectively. The highest HR for all-cause mortality (hazard ratio (HR), 1.64; 95% CI (confidence interval), 1.25-2.15), and particularly for CVD mortality (HR, 2.13; 95% CI, 1.37-3.31), was reported among individuals with severe PD, again only for younger individuals (aged 30-64 years).^4^

In the Women’s Health Study, CVD outcomes were found more often among women with PD (incidence and prevalence). The incidence rates for PD were highest for major CVD (adjusted HR, 1.42; 95% CI, 1.14-1.77), for MI ( myocardial infarction) (HR, 1.72; 95% CI, 1.25-2.38), for ischemic stroke (HR, 1.41; 95% CI, 1.02-1.95) and for total CVD (HR, 1.27; 95% CI, 1.06-1.52).

Furthermore, recent studies reported positive associations between periodontal disease and subclinical atherosclerosis, mostly reporting alterations in carotid intima-media thickness among individuals with PD.^6^^,^^7^^,^^8^The inflammation process of PD may trigger systemic inflammation that could be involved in the progression of atherosclerosis and consequently in CVD outcomes such as myocardial infarction or stroke.^9^ However, it is not totally clear what the causal pathway between PD and CVD risk is.^10^ Many observational studies have evaluated the association between different levels of periodontal disease and traditional CVRFs, such as obesity or correlated measurements,^11^^,^^12^^,^^13^^,^^14^^,^^15^^,^^16^^,^^17^^,^^18^^,^^19^^,^^20^^,^^21^ while others have reported some association with diabetes^22^ or metabolic disorders.^23^^,^^24^ Among these studies, most reported an increased odds ratio (OR) for the relationship between obesity and periodontal disease.^13^^,^^15^^,^^17^^,^^18^^,^^19^^,^^20^^,^^21^

Despite the importance of PD as a potential emerging cardiovascular risk,^25^^,^^26^ there is a paucity of systematic data that might justify inclusion of dental examination as a screening strategy for intermediate to high-risk individuals such as those presenting obesity or diabetes and those with a previous family history of CHD.^1^^,^^27^

Moreover, there are no studies that included comprehensive dental examination, including data on oral hygiene and cardiovascular risk factor evaluation at the same time, in a young to middle-aged population. Such populations probably present the greatest susceptibility to CVD risk in the presence of PD.^2^^,^^4^

## OBJECTIVE

We aimed to appraise the relationship between periodontal disease (gingivitis and periodontitis) and traditional CVRFs (obesity, hypertension, dyslipidemia, diabetes and metabolic syndrome) among young and middle-aged adults who were attended at a health promotion and check-up center in the city of São Paulo, Brazil.

## METHODS

### Study design and sample

This was a cross-sectional study conducted on a consecutive sample of all consecutive young and middle-aged individuals who were free from CVD (myocardial infarction, coronary revascularization, heart failure and stroke) and who sought attendance through a health promotion program between February and November 2012. All of them underwent standard cardiovascular and dental screening. It should be noted that the normal attendance rate at this check-up center is approximately 300 adults older than 18 years of age per month.

The Health Promotion and Check-up Center forms part of the complex of Hospital Sírio-Libanês (Syrian-Lebanese Hospital), which is a tertiary-level private hospital located in the central zone of the city of São Paulo, one of most populous cities and the most important financial center of Brazil. The population regularly attended at this check-up center is mainly composed of active middle-aged male workers.

The study protocol was approved by the Institutional Review Board that addresses research on human participants, in accordance with the Declaration of Helsinki. All the participants signed an informed consent form.

### Data-gathering

All individuals underwent a standard evaluation focusing on cardiological assessment and clinical examinations that were based on screening strategies for the general population recommended by the United States Preventive Services Task Force and the Centre for Health Promotion Canada.^28^^,^^29^

Sociodemographic data such as age (mean ± standard deviation) and educational level (up to completed high school or completed undergraduate course), data on CVRFs (hypertension, dyslipidemia, diabetes, obesity and metabolic syndrome) and lifestyle information, which included smoking status (never, former and current) and alcohol consumption (at least once a week), were all evaluated by cardiologists who were specialists in CVD screening. The screening was done in accordance with standardized protocols developed by the check-up center.

### Oral health examination

Five dentists at our check-up center, who had been trained by a specialist in periodontal diseases, were responsible for replicating a standardized protocol for oral examinations, in order to maintain the homogeneity of the data gathered.

Information on oral hygiene was obtained using a questionnaire that asked for the following data: frequency of toothbrushing, use of dental floss and presence of halitosis. Oral hygiene was categorized into three levels as follows: poor (0-1 toothbrushing/day + 0-1 dental flossing/day + presence or absence of halitosis; score 0-3); moderate (2 toothbrushings/day + 0-2 dental flossings/day + presence or absence of halitosis; score 4-5); or good (≥ 3 toothbrushings/day + ≥ 3 dental flossings/day + absence of halitosis; score ≥ 6).

All the individuals screened also underwent an oral evaluation using a periodontal probe to measure pocket depths around each tooth, in order to establish the state of health of the periodontium. All teeth were examined at six different sites (mesiobuccal, mediobuccal, distobuccal, mesiolingual, mediolingual and distolingual). All sites were probed and a pocket was considered to be present when the probing depth was 4 mm or greater at at least one site. Periodontal disease outcomes were categorized as follows:

1. Gingivitis: defined as gingival bleeding after 10 seconds of probing;
2. Periodontitis: defined as presence of four or more teeth with one or more sites with probing pocket depth greater than or equal to 4 mm and clinical attachment loss ≥ 3 mm; and
3. Absence of periodontal disease (reference group): no signs of inflammation or pocketing.^30^

Based on this oral examination, each individual was also classified as having poor, fair, good or excellent oral health.

### Cardiovascular risk factor definition

Hypertension was defined from the mean of the latest two systolic and diastolic blood pressure (BP) measurements, made using the Omron HEM 705CP oscillometric device. Three measurements were made, at one-minute intervals. Furthermore, the definition of hypertension included previous use of medication to treat hypertension, and systolic BP ≥ 140 mmHg, or diastolic BP ≥ 90 mmHg.

Dyslipidemia was defined as low-density lipoprotein-cholesterol (LDL) ≥ 130 mg/dl or use of cholesterol-lowering medications. The LDL-cholesterol level was calculated using the Friedewald equation, except for cases with elevated triglyceride levels, when an enzymatic colorimetric assay was used (ADVIA 1200, Siemens). Total high-density lipoprotein-cholesterol (HDL) and ­triglycerides were analyzed by means of the enzymatic colorimetric assay (ADVIA 1200, Siemens). Ultra-sensitive C-reactive protein (us-CRP) was measured using immunochemistry (­nephelometry, Siemens).

Diabetes was defined as a medical diagnosis or use of medication to treat diabetes, or was based on fasting plasma glucose level ≥ 126 mg/dl or glycated hemoglobin (HbA1C) ≥ 6.5%.

Body mass index (BMI) was calculated by dividing weight in kilograms by height in meters squared. Obesity was defined as BMI ≥ 30 kg/m^2^.

Metabolic syndrome was defined in accordance with the NCEP ATP III criteria (Third Report of National Cholesterol Education Program Expert Panel on Detection, Evaluation and Treatment of High Blood Cholesterol in Adults),^31^ as the presence of three of the following items: waist measurement > 88 cm for women or 102 cm for men; HDL-cholesterol < 50 mg/dl for women or < 40 mg/dl for men; systolic blood pressure ≥ 130 mmHg or diastolic blood pressure ≥ 85 mmHg; serum triglyceride levels ≥ 150 mg/dl; and fasting plasma glucose ≥ 100 mg/dl (National Cholesterol Education Program Expert Panel on Detection, Evaluation and Treatment of High Blood Cholesterol, 2002).

Venous blood samples were obtained after 12 hours of overnight fasting. The serum obtained after centrifugation was used for hormone and biochemical measurements. Analyses were carried out using an automated analyzer.

### Statistical analysis

Baseline characteristics and CVRF and laboratory data were analyzed in accordance with periodontal disease status (absence of periodontal disease or presence of gingivitis or periodontitis). Categorical variables were expressed as proportions and compared using the chi-square test. Continuous variables were expressed as means (with standard deviation) and compared using analysis of variance (ANOVA), or as medians (with range) using the Kruskal-Wallis test, according to the distribution of the variables. Multinomial logistic regression models were built using periodontal disease status as the dependent variable to evaluate its relationship with each CVRF (hypertension, diabetes, obesity and metabolic syndrome). From this, the OR with its respective 95% CI was presented in the following models: model 1 (crude); model 2 (adjustment for age and sex); model 3 (adjustment for age, sex, smoking and current alcohol consumption); and model 4 (adjustment for age, sex, smoking and current alcohol consumption and oral hygiene).

The analyses were performed using the Statistical Package for the Social Sciences (SPSS) version 22.0. For all analyses, a P-value of < 0.05 was considered significant.

## RESULTS

Table 1 shows the baseline characteristics and the distribution of CVRFs among the 539 adults who were screened at the check-up center during the study period, according to their periodontal disease status. In this population, the mean age was 45 years (± SD, 8.8), most of the participants were male (82%) and the educational level was high (97% with college or university degree). It was found that 63.2% presented periodontal disease (50.6% with gingivitis and 12.6% with periodontitis). Individuals with periodontitis were three years older than those without this condition (P < 0.001).

**Table 1:**
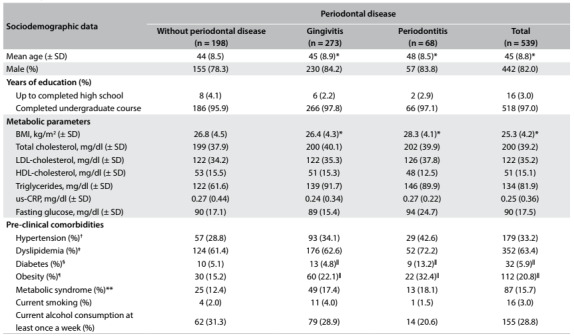
Baseline characteristics of 539 healthy young and middle-aged adults at a Brazilian health promotion and check-up center, according to periodontal disease

Some proportions might not add up to 100%, due to rounding or missing values (at most 4.7%). *P-value < 0.001 compared with controls (without gingivitis); ^†^Hypertension was defined as systolic blood pressure ≥ 140 mmHg or diastolic blood pressure ≥ 90 mmHg, history of hypertension diagnosed by a physician, or current treatment; ^‡^Diabetes was defined as previous medical history of diabetes, use of medication to treat diabetes, fasting plasma glucose ≥ 126 mg/dl, 2-hour plasma glucose ≥ 200 mg/dl, or HbA1C ≥ 6.5%; ^§^Dyslipidemia was assessed in accordance with the National Cholesterol Program-Adult Treatment Panel III (NCP ATP III) guidelines^31^ as follows: LDL-cholesterol ≥ 130 mg/dl or use of lipid-lowering drug; ^||^P-value < 0.05 compared with controls; ¶Obesity: BMI ≥ 30 kg/m^2^; **Metabolic syndrome was also defined in accordance with NCEP ATP III.^31^ BMI = body mass index; LDL = low-density lipoprotein; HDL = high density lipoprotein; us-CRP = ultra-sensitive C-reactive protein Reference group: without periodontal disease.

Approximately 70% of our sample had BMI greater than or equal to 25 kg/m^2^ and obese individuals accounted for 20.8%. Other frequently observed CVRFs included dyslipidemia (62.5%), hypertension (33.2%), metabolic syndrome (15.4%) and diabetes (5.9%).

We found progressively higher BMI (P < 0.009) and higher frequencies of obesity among individuals with PD (gingivitis: 22.1%; periodontitis: 32.4%), compared with individuals without PD (15.2%), P < 0.05. We also noticed higher frequency of diabetes among adults with periodontitis (13.2%) than among those without PD (5.1%) (P < 0.05). The frequency of PD did not differ among current smokers (3.0%), former smokers (7.2%) and never smokers (89.8%). Other CVRFs, as well as laboratory parameters such us-CRP levels, were not significantly different between individuals with PD and those without this condition (Table 1).

Although 88.1% of the adults screened reported having good oral hygiene habits, which included brushing frequency of at least three times a day (84.3%), dental flossing frequency of at least once a day (66.3%) and low frequency of halitosis (1.3%), only 25.9% were considered to have good or excellent oral health. As expected, oral health was progressively worse among individuals with periodontal disease, and the frequencies of poor oral health were 25.4%, 11.9% and 1.0% among individuals with periodontitis, with gingivitis and without PD, respectively (P < 0.001). In addition, poor oral hygiene (score 0-3) was more frequent among those with periodontitis (21.2%) than among those with gingivitis (13.5%) or without PD (6.4%) (P < 0.001).

Among all the comorbidities, obesity (yes, 12.5%, versus no, 5.0%; P = 0.001) and dyslipidemia (yes, 7.7%, versus no, 4.5%; P = 0.001) were significantly associated with poor oral health. It should be noted that we found the same trend of poor oral hygiene among obese individuals, mainly due to lack of dental flossing, in comparison with non-obese individuals (frequencies of 43.8% and 31%, respectively; P = 0.06).

In our regression analyses, obesity was the only emerging risk factor that was consistently associated with pre-existing periodontitis (multivariate OR, 2.36; 95% CI, 1.23-4.52), even after adjusting for age, sex, smoking and alcohol intake, but not with gingivitis. However, after additional adjustment for oral hygiene score (brushing and dental flossing and the presence of halitosis), this finding was no longer significant (multivariate OR, 1.63; 95% CI, 0.79-3.37). The associations with periodontitis, hypertension and diabetes presented increased OR in crude analyses, but they lost their significance after progressive adjustments for multiple confounders (Table 2). Additional adjustment for oral health instead of oral hygiene did not alter the directions or significance of our findings (data not shown).

**Table 2:**
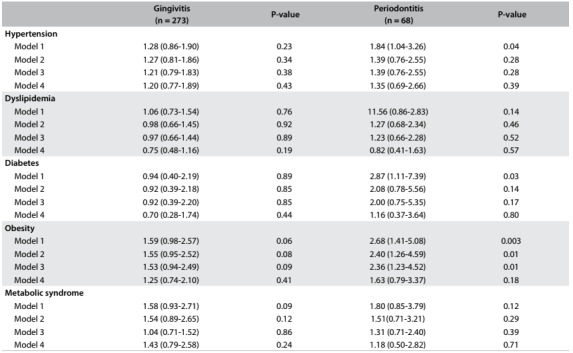
Odds ratio (with 95% CI) for the relationship between periodontal disease and cardiovascular risk factors among 539 healthy young and middle-aged adults at a Brazilian health promotion and check-up center, according to periodontal disease

Model 1 = crude; Model 2 = adjustment for age and sex; Model 3 = additional adjustment for smoking and current alcohol consumption (at least once a week); Model 4 = additional adjustment for oral hygiene. OR = odds ratio; CI = confidence interval.

## DISCUSSION

Overall, we found relatively high frequencies of periodontal disease (more than 60%) in our comprehensive screening program, which comprised dental evaluation in conjunction with cardiovascular assessment among young and middle-aged adults, in relation to other populations of the same age.^15^^,^^26^^,^^32^^,^^33^ We did not find any classical cardiovascular risk factors that were independently associated with periodontal disease. Obesity was the risk factor most closely associated with periodontitis, but after multivariate adjustment including oral hygiene and health, this risk was no longer significant.

It is well known that moderate to severe risk of having chronic morbidities can be associated with BMI levels greater than 30 kg/m^2^. Despite the heterogeneity of the studies included in a recent systematic review, a pooled OR of 1.81 (95% CI, 1.42-2.30) was reported for the relationship between obesity and periodontitis.^34^ Although several studies have reported the relationship between obesity and periodontal disease,^11^^,^^13^^,^^14^^,^^15^^,^^17^^,^^18^^,^^19^^,^^20^^,^^21^there is no consensus about which pathophysiological mechanism could explain this relationship. Most findings have come from studies with different methodologies for classifying periodontal disease, or with cross-sectional designs or selected or small samples.^11^^,^^13^^,^^14^^,^^17^^,^^18^^,^^19^^,^^20^ Some studies were conducted on large and more representative cohorts but still with diverse methodology, particularly relating to the diagnosis of PD, and were restricted to certain sex or age strata.^5^^,^^12^^,^^15^^,^^21^

Very few studies other than ours have considered oral health or hygiene status in their analyses.^12^^,^^17^ One of these was a Brazilian population-based birth cohort with a representative sample of 720 subjects in which the association between periodontal disease was determined through oral examination (at the age of 24 years) and obesity was evaluated. Waist circumference and the number of episodes of obesity between the ages of 15 and 23 years were taken to be the main exposures. The evaluation of oral hygiene included use of dental flossing and frequency of brushing. In the crude analysis, gingivitis at two or more teeth was found to be associated with obesity (OR, 1.93; 95% CI, 1.08-3.43). However, similarly to our findings, after adjusting for other confounders, including oral hygiene, obesity was found to be no longer associated with gingivitis or periodontal pockets in that cohort.^12^ The other study that included oral hygiene in its analysis (tooth brushing) was a cross-sectional study conducted among 372 Japanese adults.^17^ In that study, a dose-response relationship with pack-years of smoking, BMI and periodontitis was described, even after adjusting for confounders.^17^ The main limitations of that study were: self-reported data and the lack of information about use of dental flossing. Of note, in our study, a poor oral hygiene habit was more associated with periodontitis. In fact, lower frequency of dental flossing was observed among obese than among non-obese individuals in our study.

The explanation for the underlying pathophysiological connection between obesity and PD may be that the adverse effects of obesity on the periodontium are mediated through pro-inflammatory cytokines and various other bioactive substances.^9^ In our sample, the total levels of us-CRP were low, with little difference between obese and non-obese adults (0.22 mg/dl versus 0.11 mg/dl, respectively; P < 0.001). Other mechanisms that could link these two conditions include the quality of diets that are rich in carbohydrates or saturated fats and/or poor oral hygiene, thereby facilitating the development of local inflammatory processes.^35^ Here, we did not evaluate the role of unhealthy diets. However, our findings suggested that poor oral hygiene, mainly due to not using dental flossing, was associated with obesity, which consequently corroborated the increased risk of periodontitis.

Regarding other CVRFs, we found higher frequencies and increased ORs for the relationship between diabetes and hypertension, but these findings were attenuated in our logistic regression analyses after adjustment for multiple confounders, including oral hygiene.

Previous studies found higher incidence or prevalence of PD, particularly in cases of severe forms of diabetes, compared with the healthy population, including in prospective studies.^36^^,^^37^^,^^38^^,^^39^ High risks for the association between diabetes and periodontitis were described in the Gila River Indian Community in Arizona, where the diabetes rates are considered to be the highest worldwide. According to that study, Indians with diabetes presented increased ORs for destructive forms of periodontitis that were around three times higher than in non-diabetics, even after multivariate adjustment, including for oral hygiene.^39^ It is possible that we failed to confirm any high-risk association between PD and diabetes in our logistic analyses because in our sample the frequencies of severe forms of periodontitis and diabetes were much lower and our patients were probably healthier than those included in previous studies.^36^^,^^37^^,^^38^^,^^39^

Regarding hypertension, most studies that evaluated the risk of PD also found positive cross-sectional associations^40^^,^^41^^,^^42^and some that included oral hygiene habits in their analysis found low ORs (at most 1.5) for the relationship.^41^^,^^42^ We also found a positive association with hypertension, but after multivariate adjustments this association was no longer significant. Some characteristics, including the distribution of CVRFs and age, may be associated with these disparities among studies.

Our study has some strength. Our findings depict unique data from a young to middle-aged Brazilian population in which both classical CVRFs and periodontal disease were evaluated through an extensive dental evaluation.

Our study has some limitations. We presented data from a cross-sectional assessment, which did not allow us to make causal inferences about the relationship between CVRFs and PD. This was not a population-based study; in fact, our sample was selected from a check-up center and thus we cannot rule out the existence of selection bias that might have compromised generalization of our findings.

## CONCLUSIONS

We did not find any significant association between periodontal disease and traditional cardiovascular risk factors in this young to middle-aged sample.
